# Effect of Ropivacaine Combined with Small Doses of Triamcinolone and Continuous Nerve Block of Unilateral Paravertebral Canal Guided by Ultrasound on Metastasis after Radical Treatment of Lung Cancer

**DOI:** 10.1155/2022/6310081

**Published:** 2022-06-29

**Authors:** Dan Xu, Wei Luo

**Affiliations:** Anesthesiology Department, The Second Hospital of Dalian Medical University, Dalian, Liaoning, China

## Abstract

Continuous nerve block of unilateral paravertebral canal is one of the most reliable and the most commonly performed techniques to control intra- and postoperative pain. It is the technique of injecting local anaesthetic alongside the thoracic vertebra close to where the spinal nerves emerge from the intervertebral foramen. This produces unilateral, segmental, somatic, and sympathetic nerve blockade, which is effective for anaesthesia and in treating acute and chronic pain of unilateral origin from the chest and abdomen. Ultrasound guidance with or without nerve stimulator has reduced the amount of local anaesthetics (LA) to achieve a successful block, which may minimize complications of continuous nerve block of unilateral paravertebral canal. Previous studies have suggested that continuous nerve block of unilateral paravertebral canal with ropivacaine combined with small doses of triamcinolone was effective to achieve sensory and motor block after lung cancer surgery. Previous studies have used a fixed large dose in different volumes and concentrations for a nerve block, infraclavicular block, axillary block, and humeral canal block. The results of these studies with the respects of onset time, successful rate, and block duration were not consistent. Various factors, including the technique used, the anatomic aspects of the injection, and the pharmacodynamics aspect of drug, may influence the results. We tested the hypothesis that 50,25,10 mg ropivacaine and 50,25,10 mg triamcinolone of three combinations of volumes and concentrations for ultrasound-guided continuous nerve block of unilateral paravertebral canal produce different effects in the aspect of survival rate. Here, the drug effect was analyzed using the cross cluster K-nearest neighbors (KNN). The whole experimentation was carried out under MATLAB environment.

## 1. Introduction

Lung cancer has been the largest cause of death for both male and female worldwide, accounting for nearly 2 million fatalities each year. Lung cancer kills over 650,000 individuals in China annually, as per the National Office for Tumor Treatment as well as Management. Cigarette smoking is the leading cause of lung disease, and tobacco users account for 80 percent to 90 percent of all cases. There have been significant geographic, ethnic, and sexual disparities in cancer rates, and some researchers indicate that sensitivity of smoking toxins could put women at an elevated lung cancer risk. When opposed to a lifelong nonsmoker, a lifelong smoker seems to have a 20- to 30-fold greater cancer risk [1]. As a result, lung cancer has been the most curable among all malignancies, and quitting smoke reduces chances after a 7-year lag time. Nevertheless, this reduced risk rarely approaches background, and lung cancer has increasingly become a problem of ex-smokers in the United States. Despite advances in treatment, 90 percent of patients with lung cancer can succumb to the illness. Lung cancer claimed the lives of 1.4 million people globally in 2019, accounting for 17.9% among all cancer cases. Nevertheless, just 11.5% of regular tobacco users acquire lung disease, implying that there might be some biological factors causing risk of these diseases.

Lung cancer incidence and mortality have been rising annually; the 6-year survival rate for phase-I and phase-II non-small cell lung cancer (NSCLC) was now 68–86 percent for phase-I and 46–70 percent for phase-II illness [2]. Local anaesthetics offer a wide range of pharmacological effects, including analgesia and antiarrhythmic. These have been used to avoid or decrease extreme pain, chronic pain, and nerve pain inside a variety of therapeutic circumstances. According to a systematic analysis of tumor surgery patients, local anaesthetic may diminish disease reappearance and enhance health outcomes. During certain dosages, local anaesthetics decrease growth, inhibit migration and invasion, and cause apoptosis. Chemotherapy seems to be a common therapeutic option for cancer patients for both main and palliative care. Many individuals' diseases do not really react to such treatment, or they react effectively at first but eventually recur [3]. It might result in higher medicine dose, which often raises side effects but does not improve health diagnosis or performance. A broad range of solid tumors are linked to drug resistance. NSCLC cells were frequently innately effective against various anticancer medicines; however, SCLC cells may develop immunity with prolonged drug delivery. Furthermore, the bulk of patients with lung cancer also have metastatic illness at the clinical stage, rendering conventional treatment options, including such surgery or radiation therapy, hard when using. As a result, gaining a deeper knowledge of the many processes driving drug resistance was critical when we are to create tactics to tackle it.

Image processing uses strategies including image clustering, picture categorization, characterization-based summation, and cluster analysis to extract relevant information from datasets. [4] Extraction is indeed a common pastime among researchers. Techniques for expressing the categorization of pictures including medical image analysis include color histograms. It is used in image enhancement, information extraction, and picture definition. Many novel picture categorization approaches have successfully been applied in artificial intelligence and machine Learning. Ultrasound imaging often known as diagnostic imaging employs high-frequency sound pulses to create images of the organs. The photos may help accurately diagnose a number of illnesses [5]. The KNN cluster approach seems to be a top predictive analytics methodology. In closest neighbor categorization, the classification is performed based on the database's associated images. If combined with picture commonalities, nearest neighbor commonalities provide a semantically valid and noise-free representation of picture consistency [6].

The primary focus of this research is to examine the effect of ropivacaine combined with small doses of triamcinolone and continuous nerve block of unilateral paravertebral canal guided by ultrasound on metastasis after radical treatment of lung cancer. Initially, the datasets of the lung cancer patients are collected and then those cases are split into inclusion group and exclusion group. Then, the combination of both ropivacaine and triamcinolone is given to those patients in 3 groups of dosages with distinct amount of weights (in mg). Next, we provide the examination of these drugs. The cross cluster KNN was employed to assess the impacts of these drugs. The performance metrics of this research are analyzed with maximum effectiveness.

The further structure of this research is divided as follows: Section 2 explains the related works and problem statement, Section 3 demonstrates this research, Section 4 provides the results and discussion, and Section 5 summarizes the whole research.

## 2. Related Works

Coagulation variables may assist in predicting outcome and tracking individual therapy response. Increased PT, FIB, and D-dimer are linked to lower outcome in advanced NSCLC [7]. They suggest that individuals on 3-drug regimen had lengthier PFS than those on 2-drug regimen. To confirm these outcomes, a large prospective investigation is required [8]. It suggests that opioid-free anaesthesia for people experiencing major thoracic operation is possible. The use of Parturient-controlled epidural analgesia (PCEA) to give ropivacaine appears to diminish cumulative ropivacaine usage in the first 48 hours, and the need for morphine as well as early postoperative pain ratings [9]. Endothelial hyperpermeability would be a key factor in the formation of pulmonary edema, a life-threatening situation that could arise as a result of hypertensive crisis, acute heart inability, or lung resection surgery [10]. For diagnostic and capture-based targeted sequencing analysis, DNA collected via Endobronchial Ultrasound-Guided Transbronchial Needle Aspiration (EBUS-TBNA) specimens was adequate. For EBUS-TBNA specimens, next-generation sequencing (NGS) outperformed traditional testing without expanding the amount of specimens. NGS may play a critical part in treatment judgment among patients with advanced lung disease when they have a full genomic sequencing of the tumor [11]. They found that both Miao and Cisplatin had exactly the same effect upon lung cancer cells and also that treating lung cancerous cells including both Miao and Cisplatin at the same time may enhance the suppression effect. This research could lead to a new approach for treating cancer. Upregulation of miR-221 throughout Cisplatin cancer tissues, as well as miR-221-induced chemotherapeutic resistance of malignant cells, was determined in [12]. Furthermore, Phosphatase and TENsin homolog (PTEN) has been shown to be a focus of miR-221, making it a major contributor to Cisplatin sensitivity for malignant cells. Misdiagnosis could lead to poor decisions for therapy. The precision of machine learning and laboratory studies in cancer detection is much beneficial in treating cancer. Microarray expression information was excessively repetitive and unclear with most categories. Thus, selecting the optimum characteristic genes in tumor assessment is crucial. The strategies must be capable of robustly selecting a set with the most informative genes from a large database [13]. This article examines the categorization of lung disease using selection of features as well as enhancement strategies [14]. The essential oils from *C. citratus* of various parts of Vietnam are investigated by Gas chromatography–mass spectrometry (GC-MS). The findings demonstrate that camphene, valerianol, and epi-*α*-muurolol could be used to distinguish essential oils in lemongrass culms from leaves. The essential oils examined were cytotoxic for lung tumor types. The essential oils induced apoptosis as well as cell cycle arrest on malignant cells, causing cytotoxicity [15]. The local anaesthetic bupivacaine reduced non-small cell lung cancer (NSCLC) development by promoting autophagy via Akt/mTOR activation. Our discovery sheds light on how bupivacaine slows the growth of NSCLC. Bupivacaine could be an antitumor option for NSCLC therapy [16]. They found that four various local anaesthetics had direct inhibitory activity on esophageal cancer cells. Local anaesthetics operate on esophageal cancer cells by inhibiting Rac1, causing mitochondrial malfunction, and raising oxidative stress and damage [17]. Researchers found that bupivacaine directly suppresses mitochondrial functions and RhoA as well as Rac1 functions, which decreases gastric tumor growth, survivability, and migration and enhances chemotherapy effectiveness [18]. Bupivacaine's methods of action appear unconnected with sodium channel blockage. This evidence supports the use of appropriate anaesthetic regimens for gastric cancer patients [19]. Our findings suggest the lazertinib has been well handled, having reactions seen often in patients with NSCLC who had both activation and Tyrosine kinase inhibitor- (TKI-) resistant mutations in epidermal growth factor receptor (EGFR). Intracranial reactions have also been found often, implying that the blood–brain barrier was effectively penetrated. Lazertinib, alone or in conjunction with other medicines, seems to have a possible therapeutic function in the diagnosis of NSCLC with EGFR T790 M mutations [20]. Regarding stage III NSCLC, the predicted dosage of radiation on immune cells has been revealed to become a huge factor of local progression-free survival (LPFS), disease-free survival (DFS), and overall survival (OS). These findings are consistent with those reported earlier in a secondary data analysis of Radiation Therapy Oncology Group (RTOG) 0617, and they independently verified estimated dose of radiation on immune cells (EDRIC) as a major prognosticator in a separate cohort. The study demonstrates that ropivacaine 50 mg as 0.25, 0.5, or 0.75% solution for interscalene brachial plexus block before arthroscopic shoulder surgery produces comparable blockade with few side effects, while 0.75% seems to be more preferable as it is associated with faster onset time [21]. Considering leukocytes' highly radioactive sensitivity, the data indicate there might be an inherent balance among raising dose of radiation to promote tumor cell death and a competing tendency of immunosuppression that negates the advantages of increased doses of radiation. To investigate the possibility and effect of modifying radiation treatment regimens to preserve the host's immune pool, more modeling and analysis of EDRIC are required.

### 2.1. Problem Statement

A postoperative nerve block of the unilateral paravertebral canal is one of the most reliable and regularly used methods, injecting the LA along the thoracic vertebra at the intervertebral foramen. Unilateral segmental somatic and sympathetic nerve block is beneficial for anaesthesia and acute and chronic chest and abdominal pain. To achieve a good block, ultrasound guidance with or without a nerve stimulator has reduced the amount of the LA. The combination of ropivacaine and mild doses of triamcinolone has been shown to effectively block sensory and motor nerves following lung cancer surgery. For a nerve block, infraclavicular block, axillary block, and humeral canal block, previous research used a fixed big dose in various volumes. These trials' results on onset time, success rate, and block duration were inconsistent.

## 3. Proposed Work

**Figure 1:**
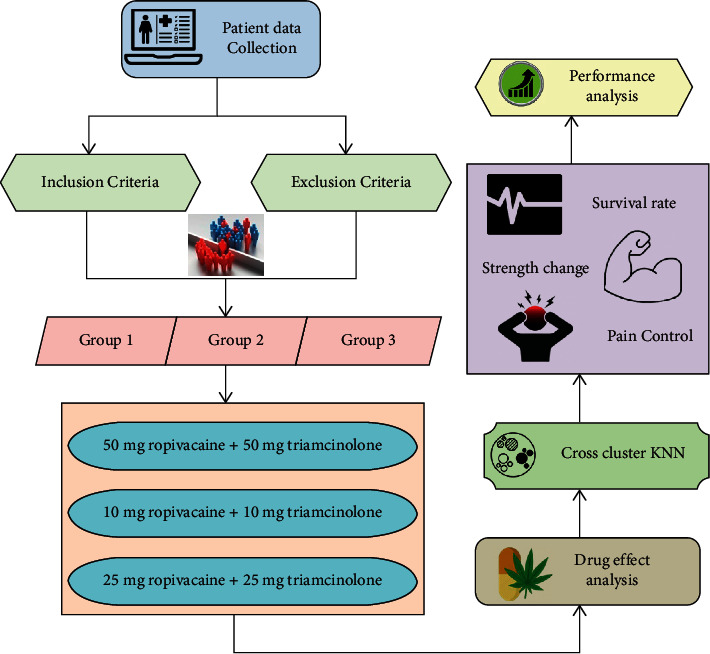
Structure of the presented methodology.

The primary focus of this research is to examine the effect of ropivacaine combined with small doses of triamcinolone and continuous nerve block of unilateral paravertebral canal guided by ultrasound on metastasis after radical treatment of lung cancer. Initially, the datasets of the lung cancer patients are collected and then those cases are split into inclusion group and exclusion group. Then, the combination of both ropivacaine and triamcinolone is given to those patients in 3 groups of dosages with distinct amount of weights (in mg). Next, we provide the examination of these drugs. The cross cluster KNN was employed to assess the impacts of these drugs.  Figure 1 depicts the complete structure of the presented methodology.

### 3.1. Patient Data Collection

The research has been approved by The Second Hospital of Dalian Medical University's Institutional Review Board (IRB). Every one of the individuals were entered into the WCLC Dataset that has been collecting data on patients with lung cancer who have had treatment at The Second Hospital of Dalian Medical University, until November 2020, and the follow-up information has been maintained.

#### 3.1.1. Inclusion Criteria

The inclusion criteria were two instances of the ICD-10 code C34 within thirty days of diagnosis although less than 365 days. When a person died within 60 days following the first C34 code, there was only one incidence of the ICD-10 C code 34. At the time of diagnosis, the patient was over the age of 20.

#### 3.1.2. Exclusion Criteria

The exclusion criteria were ICD-10 codes for cancer that are not C34 during 6 months of treatment or 12 months following treatment. During 6 months of first lung disease ICD-10 code or twelve months after the first lung cancer ICD-10 code, anticancer treatment was administered apart from lung cancer therapy procedures.

### 3.2. Group Allocation of Drug Dosage

Patients are randomly allocated to continuous nerve block of unilateral paravertebral canal with three drug dosage groups that used a factorial technique. That means the total of 108 patients receiving lung cancer treatment are randomly assigned to 3 groups:

- Group 1: 50 milligrams of the ropivacaine drug is mixed with 50 milligrams of the triamcinolone.
- Group 2: 10 milligrams of the ropivacaine drug is mixed with 10 milligrams of the triamcinolone.
- Group 3: 25 milligrams of the ropivacaine drug is mixed with 25 milligrams of the triamcinolone.

#### 3.2.1. Group 1 (50 mg ropivacaine + 50 mg triamcinolone)

In this group, 50 milligrams (mg) of ropivacaine drug was mixed with 50 milligrams of triamcinolone for dosage and that was given at the nerve block of the patients after lung cancer treating.

#### 3.2.2. Group 2 (10 mg ropivacaine + 10 mg triamcinolone)

In this group, 10 milligrams (mg) of ropivacaine drug was mixed with 10 milligrams of triamcinolone for dosage and that was given at the nerve block of the patients after lung cancer treating.

#### 3.2.3. Group3 (25 mg ropivacaine + 25 mg triamcinolone)

In this group, 25 milligrams (mg) of ropivacaine drug was mixed with 25 milligrams of triamcinolone for dosage and that was given at the nerve block of the patients after lung cancer treating.

### 3.3. Cross Cluster KNN

**Figure 2:**
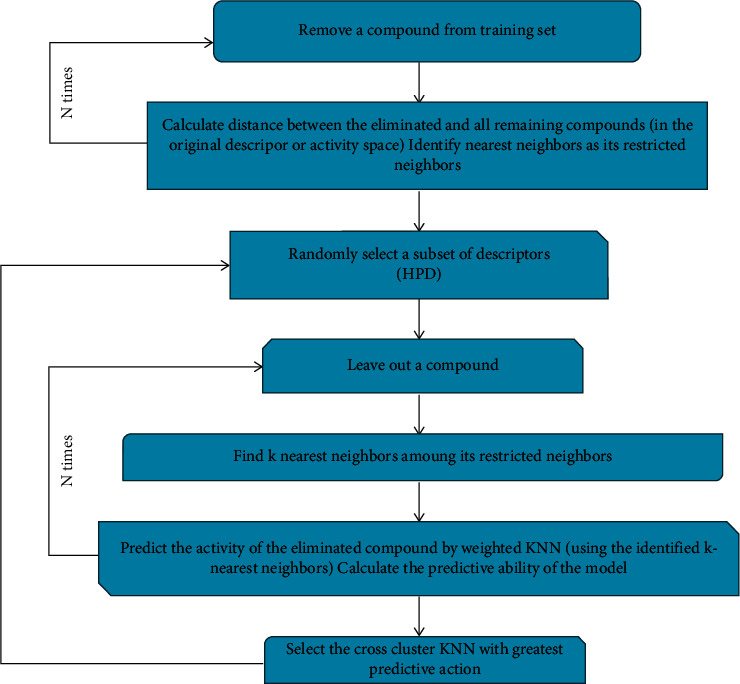
Flow of cross cluster KNN.

Figure 2 depicts the flow of the cross cluster KNN approach. Regarding variable selection, the cross cluster KNN technique uses the KNN classification concept in conjunction with the simulated annealing technique. Inside a leave-one-out cross-validation method, the action of every compound in such a drug was forecasted as the weighted mean action of its k-nearest neighbors within the drug. The approach wants to enhance multiple variables at the same time:

- the number of nearest neighbors (*n*) used to evaluate the action of every compound from authentic pool among all molecular descriptors that have been used to determine similar characteristics between compounds (i.e., ranges throughout the n-dimensional descriptor area),
- the number of nearest neighbors (*k*) applied to evaluate every compound's action,
- then *q*^2^'s score.

The procedures involved throughout the cross cluster KNN method are as follows:

  Step 1: as a hypothetical descriptor pharmacophore (HDP), choose a subgroup of n descriptors arbitrarily (*n* is indeed a value between 0 and 1 as well as the entire amount of accessible descriptors). In numerous different runs, *n* was normally assigned to varied values ranging from 10 to 50, and typically produce at least 10 models for every constant value of *n*.
  Step 2: calculate *q*^2^ for every HDP using the leave-one-out cross-validation approach given.
  Step 3: Steps 1 and 2 are repeated till we reach the highest *q*^2^ for a particular amount of *n*. Generalized simulated annealing with *q*^2^ as the target factor has been used to lead this optimization.

The following is how the typical leave-one-out technique was done:

Stage 1: remove a compound from the training drug and use the KNN approach for forecasting the target property, which would be the weighted mean characteristic of the *k* most similar molecules (*k* was fixed to 1 at the beginning):
  (1) $$\begin{array}{c} r_{i,j}=\sqrt{\sum_{k=1}^{n}{\left(D_{ik}-D_{jk}\right)}^{2}}. \end{array}$$
The Euclidean distance in between 2 compounds *i* & *j*'s descriptions throughout the descriptor area has been calculated using just the subgroup of descriptors which belongs to the present trial HDP.
Before range computations, the D descriptors created by MolConnZ are range-scaled, while no scaling is required for anteroposterior (AP) descriptors. The MolConnZ descriptors were scaled because their actual ranges differed greatly, perhaps by orders of magnitude, throughout contrast to AP descriptors whose numbers ranged from 0 to a few dozens of AP values. Therefore, while performing range computation in multidimensional MolConnZ descriptor area, the scaling is created to avoid providing descriptors with much higher ranges a higher weight.
Under this research, the classic KNN approach has been improved by employing weighted molecular similarity instead of algebraic average, as shown below. The action of every compound is forecasted using the classic technique as the algebraic average action of its *k* closest compounds throughout the training drug. Nevertheless, because the Euclidean distances between a compound and every one of its k-nearest neighbors inside the specified descriptor area would not be equal, the neighbor with the lower range from the compound is assigned a greater weight in computing the forecasted action, as illustrated in the following equation:
  (2) $$\begin{array}{c} w_{i}=\frac{\exp\left(-r_{j}\right)}{\sum_{k-\text{nearest neighors}}\exp\left(-r_{j}\right)},\\ \hat{m}=\sum^{}w_{i}m_{i}. \end{array}$$
Here, *r*_*j*_ is the Euclidean range between a compound and its ith neighbor, *i*, *w*_*i*_ is the weight of the ith neighbor, *m*_*i*_ is the real practical action value of ith neighbor, and $\hat{m}$ is the forecasted action value of the compound.
Stage 2: the first stage should be repeated until each compound inside the training drug has been eliminated and its action forecasted at least once.
Stage 3: estimate the leave-one-out cross-validated value by utilizing
  (3) q2=1−∑mi−mi^2∑mi−m^2.
These summations apply to the entire training drugs of compounds.
Stage 4: Stages 1 to 3 should be repeated with *k* = 2, 3, 4, and so on. The entire amounts of compounds inside the set of drugs represent the maximum value of *k*; nevertheless, the better value was shown experimentally to be from 1 to 5. Again for the cross cluster KNN approach, the value of *k* which corresponds to the greatest *q*^2^ score is picked.

## 4. Result and Discussion

In this phase, we are going to examine the effect of ropivacaine combined with small doses of triamcinolone and continuous nerve block of unilateral paravertebral canal guided by ultrasound on metastasis after radical treatment of lung cancer. The findings of this research are depicted by utilizing the MATLAB tool. Analytical importance has been defined as a *P* value of less than 0.05.  Table 1 depicts the statistical analysis of this research. In this investigation, we consider 3 clinical locations that are Dalian, Shenyang, and Jinzhou. For the first group, 52 cases are taken into account. Similarly, for the second and third groups, 54 and 53 patients are taken correspondingly.

For each hospital, the patients are divided with distinct numbers from those groups. That means that the first group of patients are tested in those three clinics (26 patients for Dalian clinic, 7 patients for Shenyang clinic, and 19 patients for Jinzhou clinic). The second group of patients are tested with 27 patients for Dalian clinic, 10 patients for Shenyang clinic, and 17 patients for Jinzhou clinic. The third group of patients are tested with 24 patients for Dalian clinic, 8 patients for Shenyang clinic, and 21 patients for Jinzhou clinic. Patients in this group are expected to be older than 36 years old, with men and daily smokers accounting for the majority.

All patients are tested after treatment of the lung cancer. Arthroscopic procedure type was utilized, mostly for the second group. Those groups of patients were treated mostly with rotator cuff repair procedure. That means that 25 patients had rotator cuff repair procedure, 11 patients had arthroplasty procedure, and 16 patients had other type of procedure in the first group.

**Figure 3:**
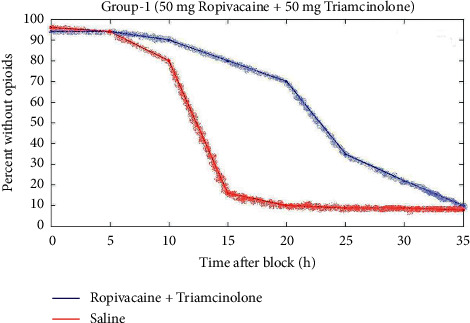
Comparison of block duration of triamcinolone with ropivacaine for group 1.

**Figure 4:**
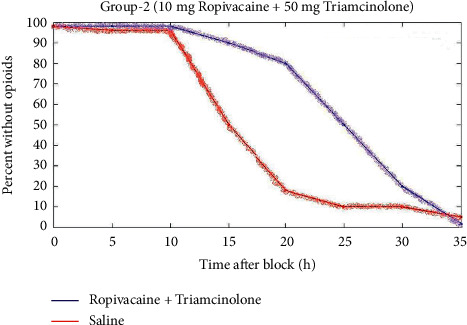
Comparison of block duration of triamcinolone with ropivacaine for group 2.

**Figure 5:**
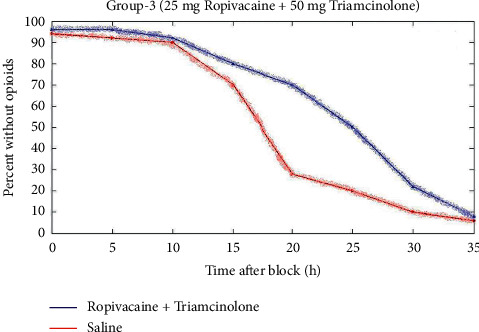
Comparison of block duration of triamcinolone with ropivacaine for group 3.

In the first group, 36 patients were utilized for ultrasound-guided technique. For the same technique, 39 patients from the second group and 42 patients from the third group were treated. And also 42 patients had nerve simulator technique used. 47 patients from the second group and 32 patients from the third group were utilizing the same nerve simulator technique. The technique was successfully performed on the first group of patients without failure of the block over other two groups. That means that there is better outcome in the first group of patients. Figures 3–5 depict the comparison of the block timespan of triamcinolone with ropivacaine for groups 1 to 3.

Figure 3 represents the comparison of block duration of triamcinolone with ropivacaine for group 1. When compared to the saline, the ropivacaine 50 mg with triamcinolone 50 mg has 41 h timespan.

Figure 4 represents the comparison of block duration of triamcinolone with ropivacaine for group 2. When compared to the saline, the 10 mg ropivacaine with 50 mg triamcinolone has 36 h timespan.

Figure 5 represents the comparison of block duration of triamcinolone with ropivacaine for group 3. When compared to the saline, the 25 mg ropivacaine with 50 mg triamcinolone has 39 h timespan.

Ropivacaine considerably increased the duration of triamcinolone analgesia. The block resolution percentage between patients given triamcinolone with ropivacaine was approximately 0.17 times [as shown in Figure 3] that of patients who received triamcinolone separately, according to segmented Cox's analysis for duration to the first opioid usage.

In triamcinolone, the impact of ropivacaine upon block durability has been significantly more powerful. When the primary outcome of block length was examined utilizing ultrasound or nerve stimulation, the technology seemed to have no discernible influence on the primary endpoint. The Kaplan–Meier graph estimations for mean block time in ultrasound-guided individuals were 12.3 versus 22.4 h with triamcinolone. The median predictions for individuals receiving nerve stimulation-guided blocking are 11.8 vs. 21 h with triamcinolone.

Our findings show that ropivacaine enhances the analgesic impact of ordinary triamcinolone and when using it as a single-injection interscalene block, and therefore this impact varies among these local anaesthetics. The result was essentially consistent with earlier research, although direct relationships are challenging due to the wide range of local anaesthetic formulations employed, the varied blocks investigated, and the diverse methodologies for determining block period.

**Figure 6:**
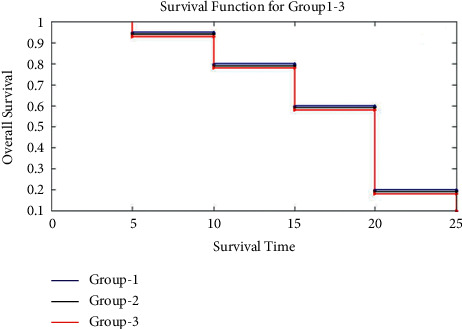
Overall survival of such patients having lung cancer for groups 1 to 3.

Figure 6 depicts the overall survival of such patients having lung cancer who are treated with the drug dosage in the distinct quantities. Survival rate of those treated patients having drug dosage can be described as follows: in order to arrive at this statistic, divide the proportion of individuals with the lung cancer who are still surviving after a certain amount of time by the proportion of members of the same gender and age who are still surviving after the same amount of time.

Figure 6 represents the overall survival of such patients having lung cancer for groups 1, 2, and 3. In overall survival, the period of survival of group 1 is 30 h, the period of survival of group 2 is 30 h, and the period of survival of group 3 is 29 h.

## 5. Conclusion

Examination of the effect of combination of ropivacaine and small doses of triamcinolone and continuous nerve block of unilateral paravertebral canal guided by ultrasound on metastasis after radical treatment of lung cancer was the primary focus of this research. Initially, the datasets of the lung cancer patients were gathered and then those cases were separated into inclusion group and exclusion group. Then, the combination of both ropivacaine and triamcinolone was given to those patients in 3 groups of dosages with distinct amount of weights (in mg). Next, the cross cluster KNN was employed to assess the impacts of these drugs after the drug assessment. The performance metrics such as survival rate of this research were analyzed with maximum effectiveness. From the results, group 1 patients with lung cancer had a 30-hour survival time, group 2 patients had a 30-hour survival period, and group 3 patients had a 29-hour survival period. With the rising frequency of lung cancer, more and more aggressive operations are being performed. Different approaches to anaesthesia and narcotics may have varying impacts on the prognosis of patients receiving lung cancer radical surgery. The use of ultrasound technology in the area of anaesthesia has allowed paravertebral nerve blocks to be administered under ultrasound guidance in recent years. Several variables influence lung tumor spread and recurrence, and studies show that anaesthetic medicines influence cancer metastasis. The selection of anaesthetic procedures and the appropriate use of anaesthetic medicines may improve anticancer effects while lowering the risk of tumor recurrence and metastasis.

## Figures and Tables

**Table 1:** Analysis of data.

Feature	50 mg ropivacaine plus 50 mg triamcinolone (*n* = 52)	10 mg ropivacaine plus 10 mg triamcinolone (*n* = 54)	25 mg ropivacaine plus 25 mg triamcinolone (*n* = 53)
Hospital location			
Dalian	26	27	24
Shenyang	7	10	8
Jinzhou	19	17	21
Age (years)			
<36	18	16	24
≥36	34	38	29
Gender			
Male	39	42	38
Female	13	12	15
Ethnicity			
Caucasian	47	51	45
Procedure type			
Arthroscopic	22	23	19
Procedure			
Rotator cuff repair	25	31	34
Arthroplasty	11	12	4
Other	16	11	15
Failed block	0	2	5
Ultrasound-guided	36	39	42
Nerve stimulator used	42	47	32

## Data Availability

The datasets used during the present study are available from the corresponding author up on request.
